# Risk factors and prediction model for mortality risk in older adults septic patients

**DOI:** 10.3389/fmed.2026.1867494

**Published:** 2026-09-01

**Authors:** Shao-Zhen He, Lu-Zhen Qiu, Yong-Xiang Li, Zhao-Bin Yang, Xiao-Mei Cheng

**Affiliations:** 1 Department of Medical Intensive Care Unit, Zhangzhou Affiliated Hospital of Fujian Medical University, Zhangzhou, Fujian, China; 2 Department of Internal Medicine, Zhangzhou Xiangcheng Hospital of Traditional Chinese Medicine, Zhangzhou, Fujian, China

**Keywords:** 28-days-mortality, prediction model, retrospective study, risk factors, sepsis

## Abstract

**Background:**

The early identification of septic patients at high risk of mortality is essential for improving clinical outcomes. This study aimed to investigate the risk factors associated with 28-day mortality among older adults septic patients admitted to the intensive care unit (ICU) and to develop a predictive model based on these factors.

**Methods:**

A total of 165 older adults septic patients admitted to the ICU of our hospital between April 2022 and March 2025 were included in this study. Multivariate logistic regression analysis was employed to identify independent risk factors associated with mortality. A prediction model was subsequently constructed and evaluated using receiver operating characteristic (ROC) curve analysis, with the area under the curve (AUC) used to assess its predictive accuracy.

**Results:**

Among the 165 septic patients, 59 experienced 28-day mortality. Multivariate logistic regression analysis indicated that higher hemoglobin levels were associated with a reduced risk of 28-day mortality (odds ratio [OR]: 0.961; 95% confidence interval [CI]: 0.938–0.985; *p* = 0.001). A simplified risk estimation approach based on hemoglobin levels demonstrated good discriminatory performance (AUC: 88.3%; 95% CI: 82.7–93.9%). However, given that hemoglobin was the sole independent predictor retained in the multivariable model, the predictive performance should be interpreted as reflecting the prognostic value of this single biomarker rather than a true multivariable risk model. The findings should be considered exploratory and hypothesis-generating, requiring external validation in larger cohorts.

**Conclusion:**

This exploratory study identified hemoglobin as a potentially useful biomarker for risk stratification in older septic patients. A simplified risk estimation approach based on hemoglobin demonstrated good preliminary discriminatory performance, but these findings should be interpreted with caution and require external validation in larger cohorts before clinical application.

## Introduction

Sepsis arises from a dysregulated host response to infection, characterized by an overwhelming systemic inflammatory reaction and a high risk of mortality (1). Each year, millions of individuals worldwide are affected by sepsis and septic shock, presenting a significant burden on global healthcare systems (2). A particularly pressing issue is the persistently high mortality rate among these patients, with 28-day mortality serving as a key endpoint that reflects disease severity and clinical outcomes (3). This challenge is compounded by several factors, including advanced age, impaired immune function, the rising incidence of multidrug-resistant infections, and delays in diagnosis and treatment (4, 5). It is estimated that approximately 3% of all hospitalized patients are diagnosed with sepsis, about half of whom require admission to intensive care units (ICUs). Despite advances in critical care, the 28-day mortality rate in this population remains notably high, often exceeding 30% (6). Elderly individuals are especially vulnerable due to age-related immunosuppression and higher susceptibility to severe infections, leading to worse clinical outcomes (5). In fact, older adults aged 65 and above account for 60–85% of all severe sepsis cases, a proportion that is anticipated to rise alongside the growing geriatric population worldwide (7). Therefore, the early and accurate identification of risk factors associated with 28-day mortality in septic patients—particularly among the older adults—is of paramount importance. Developing robust predictive models not only aids in risk stratification but also supports timely clinical decision-making, ultimately helping to improve prognosis in this high-risk group.

Current prognostic models in sepsis care are predominantly designed to evaluate disease severity and predict general clinical outcomes. These models widely adopt established clinical scoring systems, such as the Sequential Organ Failure Assessment (SOFA), Quick Sequential (Sepsis-Related) Organ Failure Assessment (qSOFA), Acute Physiology and Chronic Health Evaluation II (APACHE II), Oxford Acute Severity of Illness Score (OASIS), and Simplified Acute Physiology Score II (SAPS II) (8, 9). While existing prognostic tools such as SOFA and APACHE II are widely used, they are not specifically tailored to older adults septic patients, and few incorporate routinely available laboratory parameters such as hemoglobin and coagulation markers. Moreover, most predictive models in sepsis have been developed in mixed-age populations, leaving a gap in tools optimized for older adults, who exhibit distinct physiological responses and higher susceptibility to adverse outcomes. This study seeks to fill this gap by systematically identifying independent risk factors associated with 28-day mortality in older adults ICU patients with sepsis and developing a simplified risk estimation approach to support early risk stratification. We emphasize that this analysis is exploratory in nature and that the proposed approach should not be considered a validated clinical prediction tool until it has undergone external validation in independent cohorts.

## Materials and methods

### Study design and patients

A retrospective cohort study was conducted involving 165 older adults septic patients admitted to the intensive care unit (ICU) of our hospital between April 2022 and March 2025. Inclusion criteria were as follows: (1) age ≥55 years. While the conventional definition of ‘older adults’ in geriatric medicine often uses a threshold of 65 years, we chose an age threshold of ≥55 years based on several considerations. First, accumulating evidence suggests that age-related physiological decline in immune function and organ reserve begins earlier than traditionally recognized, particularly in the context of sepsis (10, 11). Second, the incidence and mortality of sepsis increase progressively with age, with a notable inflection point observed around age 55 in epidemiological studies (12). Third, using a lower threshold allowed us to include a more representative spectrum of older patients in our cohort, as our hospital serves a population with limited access to geriatric specialty care; (2) diagnosis of sepsis based on the “Sepsis-3” international consensus criteria (2016), defined as life-threatening organ dysfunction caused by a dysregulated host response to infection, with an acute increase of ≥2 points in the SOFA score from baseline (13); (3) ICU hospitalization exceeding 24 h; and (4) availability of complete clinical and 28-day follow-up data. Exclusion criteria comprised: (1) age <55 years; (2) length of ICU stay <24 h; (3) pregnancy; (4) clinical records with >25% missing data; and (5) transfer to another facility within 72 h of ICU admission. These exclusions were applied to minimize confounding factors and ensure robust assessment of predictors related to 28-day mortality. The study protocol was approved by the Institutional Review Board of Zhangzhou Affiliated Hospital of Fujian Medical University (Approval No: 2024LWB372). Due to the retrospective nature of the research, the requirement for informed consent was waived.

### Variable and outcome definition

Based on clinical relevance and current evidence in the literature, the following variables were comprehensively extracted from the electronic health records: (1) Baseline demographic characteristics: sex and age; (2) Comorbid conditions: including hypertension, diabetes, cardiovascular diseases, and history of stroke; (3) Disease severity assessment: the APACHE II score, evaluated within the first 24 h following ICU admission (incorporating acute physiological parameters, age, and chronic health status); (4) Laboratory parameters (measured within 24 h of ICU admission): (1) Inflammatory indicators: white blood cell count (WBC), neutrophil percentage, procalcitonin (PCT), C-reactive protein (CRP); (2) Hematological indicators: platelet count (PLT), hemoglobin (HGB); (3) Coagulation and fibrinolysis indicators: prothrombin time (PT), international normalized ratio (INR), activated partial thromboplastin time (APTT), fibrinogen (Fib), D-dimer (D-D), fibrin degradation products (FDP), thrombin–antithrombin complex (TAT), thrombomodulin (TM), plasminogen activator inhibitor-1 (PAI-1), tissue-type plasminogen activator–plasminogen activator inhibitor complex (tPAIC), and plasminogen activator inhibitor complex (PIC); (5) Treatment-related variable: administration of heparin therapy during the ICU stay; (6) hospital length of stay before ICU admission (hours); (7) adequate antibiotic therapy within 6 h was defined as administration of empiric antibiotics that: (1) covered the most likely pathogens based on infection site, local antibiogram, and patient risk factors; and (2) were subsequently confirmed to be active against the identified pathogen(s) by susceptibility testing, or appropriately adjusted within 24 h of susceptibility results. For culture-negative cases, therapy was considered adequate if it followed established guidelines and the patient showed clinical improvement. Two independent infectious disease physicians adjudicated all cases, with disagreements resolved by consensus; (8) requirement for source control; (9) presence of septic shock (defined as vasopressor requirement to maintain mean arterial pressure ≥65 mmHg despite adequate fluid resuscitation and serum lactate >2 mmol/L). Organ support received during the first 24 h of ICU admission was recorded, including: (1) mechanical ventilation (invasive or non-invasive); (2) vasopressor therapy (any dose of norepinephrine, dopamine, or vasopressin); (3) renal replacement therapy (continuous or intermittent). The primary outcome of interest was 28-day all-cause mortality following ICU admission, defined as death from any cause within 28 days after the diagnosis of sepsis.

### Statistical analysis

Given the sample size and the number of candidate variables, a *post hoc* power analysis was conducted. With an event rate of 35.8% (59 deaths), the study achieved approximately 80% power to detect an odds ratio of 1.5 for a continuous predictor at an alpha level of 0.05, suggesting adequate but not robust power for the primary analysis. Nevertheless, the risk of overfitting remains, and findings should be considered hypothesis-generating. Missing data patterns are summarized in Supplementary Table S1. Variables with <15% missingness were imputed using multiple imputation by chained equations (MICE) with 20 imputations, incorporating all variables in the analysis model, including outcome status. For specialized coagulation assays (TAT, TM, PIC, tPAIC) with >20% missingness, complete case analysis was performed as the primary approach, with sensitivity analyses.

Patient characteristics were described according to the distribution of the data. Continuous variables were summarized as mean (standard deviation) for normally distributed data or median (interquartile range) for non-normally distributed data. Group comparisons were performed using independent samples *t*-tests or Mann–Whitney U tests, as appropriate. Categorical variables were expressed as numbers (percentages) and compared using the chi-square (χ^2^) test or Fisher’s exact test, where applicable. For continuous variables, ORs are reported per clinically meaningful increments to enhance interpretability: INR per 0.1-unit, and D-D per 1,000 ng/mL. These increments were selected based on clinical relevance and the distribution of the data. Univariate logistic regression analysis was conducted to identify potential risk factors associated with 28-day mortality in septic patients. Variables with a significance level of *p* < 0.10 in univariate analysis were entered into a multivariate logistic regression model using stepwise selection (entry threshold *α* = 0.05, removal threshold *β* = 0.10). Variables representing downstream clinical consequences of sepsis severity—including septic shock, mechanical ventilation, and vasopressor use—were not included as candidate predictors in the multivariate model because they lie on the causal pathway between underlying pathophysiological processes and mortality. Adjustment for such intermediate variables can introduce overadjustment bias and obscure the prognostic value of earlier biomarkers. These variables are therefore reported descriptively and in univariate analyses but were not entered into the multivariate regression model. While conventional definitions of ‘older adults’ often use a threshold of 65 years, we chose an age threshold of ≥55 years to capture a more representative spectrum of older patients with age-related physiological decline in immune function and organ reserve, which is particularly relevant in sepsis. To validate this approach, we conducted subgroup analyses stratifying patients into age 55–64 and age ≥65 groups.

To address the risk of overfitting associated with the limited sample size and number of candidate variables, we performed a sensitivity analysis using least absolute shrinkage and selection operator (LASSO) regression with 10-fold cross-validation. The LASSO method applies a penalty to the regression coefficients, shrinking less important variables toward zero and reducing overfitting. The optimal penalty parameter (*λ*) was selected using the minimum cross-validated deviance criterion. Variables with non-zero coefficients in the LASSO model were considered to have the most robust associations. For the final multivariable model, the logistic regression equation was defined as: logit(*p*) = β_0_ + β_1_ × (variable_1_), where p represents the predicted probability of 28-day mortality. The optimal cut-off value for the predicted probability was determined using the Youden index (maximizing sensitivity + specificity − 1). Model performance metrics including sensitivity, specificity, positive predictive value (PPV), and negative predictive value (NPV) were calculated at the optimal cut-off. Discrimination was assessed using the area under the receiver operating characteristic curve (AUC-ROC). Calibration was assessed using the Hosmer-Lemeshow goodness-of-fit test and a calibration plot comparing predicted versus observed probabilities, with calculation of the calibration slope and intercept. The Brier score was calculated as a measure of overall prediction error, with values ranging from 0 (perfect prediction) to 0.25 (non-informative prediction for binary outcomes). Clinical utility was evaluated using decision curve analysis (DCA), which estimates the net benefit of using the model across a range of threshold probabilities. For internal validation, we employed both bootstrap resampling with 1,000 iterations (using optimism correction for AUC, calibration slope, and Brier score) and 5-fold nested cross-validation with 10 repetitions. In the nested cross-validation, LASSO variable selection was performed in the inner loop and performance evaluated in the outer loop to provide a more conservative estimate of the model’s generalizability. The calibration slope was calculated using logistic recalibration, and the shrinkage factor was derived from the bootstrap procedure to adjust the model coefficients for future applications. All statistical tests were two-sided, and a *p*-value < 0.05 was considered statistically significant. All analyses were performed using SPSS software (version 26.0; IBM Corp., Armonk, NY, USA).

## Results

### Baseline characteristics

A total of 165 older adults patients with sepsis admitted to the ICU were included in this study, comprising 117 (70.9%) males and 48 (29.1%) females. Among the 165 patients, 102 (61.8%) presented with septic shock. The proportion of septic shock was significantly higher in non-survivors compared with survivors (81.4% vs. 50.9%, *p* < 0.001). Regarding organ support: mechanical ventilation was required in 98 patients (59.4%), with higher rates in non-survivors (79.7% vs. 48.1%, *p* < 0.001); vasopressor therapy was used in 112 patients (67.9%), again more frequent in non-survivors (89.8% vs. 55.7%, *p* < 0.001); renal replacement therapy was used in 31 patients (18.8%), with no significant difference between groups (23.7% vs. 16.0%, *p* = 0.224). Adequate antibiotic treatment was administered in 128 patients (77.6%), and source control was required in 47 patients (28.5%). No significant differences in adequate antibiotic administration were observed between survivors and non-survivors (78.3% vs. 76.3%, *p* = 0.762). However, non-survivors more frequently required source control (37.3% vs. 23.6%, *p* = 0.048). The median pre-ICU hospital length of stay was 8 h (IQR: 3–24 h), with no significant difference between survivors and non-survivors (median 7 vs. 10 h, *p* = 0.312). The baseline characteristics of the participants are summarized in Table 1. Among these patients, 59 (35.8%) died within 28 days of admission. Significant differences between survivors and non-survivors were observed in several laboratory parameters: HGB (*p* < 0.001), PT (*p* = 0.002), INR (*p* = 0.005), FDP (*p* = 0.046), D-D (*p* = 0.009), PIC (*p* = 0.001), and tPAIC (*p* = 0.002). In contrast, no statistically significant differences were found between the two groups in terms of age (*p* = 0.074), sex (*p* = 0.295), or comorbidities including hypertension (*p* = 0.325), diabetes (*p* = 0.307), cardiovascular diseases (*p* = 0.305), and history of stroke (*p* = 0.268). Additionally, no significant differences were observed in the APACHE II score (*p* = 0.062), WBC (*p* = 0.061), neutrophil percentage (*p* = 0.844), PCT (*p* = 0.995), CRP (*p* = 0.425), APTT (*p* = 0.295), fibrinogen (*p* = 0.452), TAT (*p* = 0.543), TM (*p* = 0.349), or the use of heparin therapy (*p* = 0.138).

**Table 1:** Baseline characteristics of enrolled older septic patients stratified by 28-day mortality status.

Variable	Mortality		
	No (*n* = 106)	Yes (*n* = 59)	*p* value
Age (years)	66.20 ± 14.83	70.53 ± 14.33	0.071
Sex (male, %)	72 (68.6%)	45 (76.3%)	0.295
Hypertension (%)	35 (33.0%)	24 (40.7%)	0.325
Diabetes (%)	23 (21.7%)	17 (28.8%)	0.307
CVD (%)	9 (8.5%)	8 (13.6%)	0.305
Stroke (%)	8 (7.5%)	10 (16.9%)	0.268
APACHE score	24.01 ± 6.61	26.25 ± 8.15	0.062
Septic shock	54 (50.9%)	48 (81.4%)	<0.001
Mechanical ventilation	51 (48.1%)	47 (79.7%)	<0.001
Vasopressor use	59 (55.7%)	53 (89.8%)	<0.001
Renal replacement therapy	17 (16.0%)	14 (23.7%)	0.224
WBC (10^9^/L)	14.45 ± 8.15	12.09 ± 6.85	0.061
Neutrophil (%)	83.25 ± 13.26	83.73 ± 17.41	0.844
PLT (10^9^/L)	122.96 ± 103.97	99.49 ± 95.12	0.154
HGB (g/L)	101.48 ± 25.61	84.51 ± 18.08	<0.001
PCT (ng/mL)	22.90 ± 36.65	22.94 ± 34.37	0.995
CRP (mg/L)	113.44 ± 93.80	128.49 ± 141.47	0.425
PT (s)	15.66 ± 4.09	18.03 ± 4.72	0.002
INR	1.34 ± 0.39	1.55 ± 0.47	0.005
APTT (s)	44.20 ± 18.60	47.80 ± 22.30	0.295
Fib (g/L)	3.42 ± 1.85	3.50 ± 1.90	0.452
FDP (mg/L)	24.56 ± 33.26	38.34 ± 40.05	0.046
D-D (ng/mL)	5290.94 ± 6397.62	8948.53 ± 9242.34	0.009
TAT (ng/mL)	14.71 ± 19.83	16.54 ± 15.90	0.543
TM (TU/mL)	24.28 ± 16.08	26.71 ± 14.64	0.349
PIC (ug/mL)	6.10 ± 11.18	2.25 ± 3.62	0.001
tPAIC (ng/mL)	18.81 ± 18.86	28.87 ± 20.28	0.002
Use of heparin (%)	51 (48.1%)	22 (37.3%)	0.138

CVD, cardiovascular disease; APACHE II, Acute Physiology and Chronic Health Evaluation II; WBC, white blood cell count; PLT, platelet count; HGB, hemoglobin; PCT, procalcitonin; CRP, C-reactive protein; PT, prothrombin time; INR, international normalized ratio; APTT, activated partial thromboplastin time; Fib, fibrinogen; FDP, fibrin degradation products; D-D, D-dimer; TAT, thrombin-antithrombin complex; TM, thrombomodulin; PIC, plasminogen activator inhibitor complex; tPAIC, tissue-type plasminogen activator–plasminogen activator inhibitor complex.

### Influencing factors for sepsis

After adjusting for potential confounding factors using multivariate logistic regression, HGB was identified as a significant protective factor, with each unit increase associated with a 3.9% reduction in the risk of 28-day mortality (odds ratio [OR]: 0.961; 95% confidence interval [CI]: 0.938–0.985; *p* = 0.001). To assess the robustness of the findings, a sensitivity analysis was performed using a clinically pre-specified model including age, HGB, and PT. This model yielded similar results (HGB OR: 0.959; 95% CI: 0.937–0.982; *p* < 0.001), supporting the stability of the primary analysis. For specialized coagulation assays (TAT, TM, PIC, tPAIC) with >20% missingness, complete case analysis was performed as the primary approach, with sensitivity analyses using predictive mean matching imputation, MNAR scenario modeling, and pattern mixture models stratified by missingness status. The primary findings remained robust across all sensitivity analyses (Supplementary Table S2).

During the first 48 h of ICU admission, 24 patients (14.5%) received RBC transfusions and 8 patients (4.8%) had documented active bleeding. To assess the potential confounding effect of transfusions and bleeding on the relationship between hemoglobin and mortality, we conducted sensitivity analyses by: adjusting for transfusion status in the multivariate model (HGB OR: 0.963; 95% CI: 0.938–0.989; *p* = 0.003); excluding transfusion recipients (HGB OR: 0.958; 95% CI: 0.931–0.986; *p* = 0.003); and excluding active bleeding cases (HGB OR: 0.960; 95% CI: 0.937–0.984; *p* = 0.001). No significant interaction was observed between hemoglobin and transfusion status (*p* = 0.342). These analyses suggest that the prognostic value of hemoglobin is robust to the potential confounding effects of transfusion and bleeding. No statistically significant associations were observed between the other variables and 28-day mortality after adjustment. Specifically, the following factors did not demonstrate significant independent effects: age (OR: 1.025; 95% CI: 0.989–1.063; *p* = 0.182), sex (OR: 0.335; 95% CI: 0.109–1.027; *p* = 0.056), hypertension (OR: 1.091; 95% CI: 0.383–3.108; *p* = 0.870), diabetes (OR: 2.056; 95% CI: 0.565–7.482; *p* = 0.274), cardiovascular diseases (OR: 1.104; 95% CI: 0.230–5.311; *p* = 0.901), history of stroke (OR: 4.644; 95% CI: 0.855–25.220; *p* = 0.075), APACHE II score (OR: 1.024; 95% CI: 0.951–1.103; *p* = 0.533), WBC count (OR: 0.931; 95% CI: 0.859–1.009; *p* = 0.080), neutrophil percentage (OR: 1.020; 95% CI: 0.981–1.060; *p* = 0.326), PLT (OR: 0.999; 95% CI: 0.994–1.005; *p* = 0.798), PCT (OR: 1.004; 95% CI: 0.986–1.023; *p* = 0.639), CRP (OR: 1.002; 95% CI: 0.997–1.007; *p* = 0.375), PT (OR: 2.172; 95% CI: 0.987–4.781; *p* = 0.054), INR (OR: 0.501; 95% CI: 0.184–1.363; *p* = 0.176), APTT (OR: 1.001; 95% CI: 0.992–1.011; *p* = 0.760), Fib (OR: 0.999; 95% CI: 0.987–1.011; *p* = 0.868), D-D (OR: 1.009; 95% CI: 0.994–1.024; *p* = 0.261), TAT (OR: 1.014; 95% CI: 0.980–1.050; *p* = 0.427), TM (OR: 0.986; 95% CI: 0.945–1.029; *p* = 0.521), PIC (OR: 0.897; 95% CI: 0.782–1.030; *p* = 0.124), tPAIC (OR: 1.030; 95% CI: 0.995–1.067; *p* = 0.096), and heparin use (OR: 0.461; 95% CI: 0.161–1.327; *p* = 0.151) (Table 2). Subgroup analyses according to patients’ age found the association between hemoglobin and mortality was consistent across both subgroups (OR: 0.702 [95% CI: 0.518–0.951] vs. 0.651 [95% CI: 0.509–0.832]; P for interaction = 0.712), and the AUC was similar (86.5% vs. 89.1%), supporting the use of the broader age threshold (Supplementary Table S3).

**Table 2:** Multivariate logistic regression analysis for 28-day mortality in older septic patients.

Variable	*β*	OR (95% CI)	*p* value
Age (years)	0.025	1.025 (0.989–1.063)	0.182
Sex (male, %)	−1.094	0.335 (0.109–1.027)	0.056
Hypertension (%)	0.087	1.091 (0.383–3.108)	0.870
Diabetes (%)	0.721	2.056 (0.565–7.482)	0.274
CVD (%)	0.099	1.104 (0.230–5.311)	0.901
Stroke (%)	1.536	4.644 (0.855–25.220)	0.075
APACHE score	0.024	1.024 (0.951–1.103)	0.533
WBC (10^9^/L)	−0.072	0.931 (0.859–1.009)	0.080
Neutrophil (%)	0.019	1.020 (0.981–1.060)	0.326
PLT (10^9^/L)	−0.001	0.999 (0.994–1.005)	0.798
HGB (g/L)	−0.040	0.961 (0.938–0.985)	0.001
PCT (ng/mL)	0.004	1.004 (0.986–1.023)	0.639
CRP (mg/L)	0.002	1.002 (0.997–1.007)	0.375
PT (s)	0.776	2.172 (0.987–4.781)	0.054
INR (per 0.1-unit)	−0.691	0.501 (0.184–1.363)	0.176
APTT (s)	0.001	1.001 (0.992–1.011)	0.760
Fib (g/L)	−0.001	0.999 (0.987–1.011)	0.868
D-D (ng/mL) (per 1,000 ng/ml)	0.009	1.009 (0.994–1.024)	0.261
TAT (ng/mL)	0.014	1.014 (0.980–1.050)	0.427
TM (TU/mL)	−0.014	0.986 (0.945–1.029)	0.521
PIC (ug/mL)	−0.108	0.897 (0.782–1.030)	0.124
tPAIC (ng/mL)	0.030	1.030 (0.995–1.067)	0.096
Use of heparin (%)	−0.773	0.461 (0.161–1.327)	0.151

CI, confidence interval; CVD, cardiovascular disease; APACHE II, Acute Physiology and Chronic Health Evaluation II; WBC, white blood cell count; OR, odds ratio; PLT, platelet count; HGB, hemoglobin; PCT, procalcitonin; CRP, C-reactive protein; PT, prothrombin time; INR, international normalized ratio; APTT, activated partial thromboplastin time; Fib, fibrinogen; FDP, fibrin degradation products; D-D, D-dimer; TAT, thrombin-antithrombin complex; TM, thrombomodulin; PIC, plasminogen activator inhibitor complex; tPAIC, tissue-type plasminogen activator–plasminogen activator inhibitor complex.

To address the risk of overfitting associated with the limited sample size, we performed a sensitivity analysis using LASSO regression with 10-fold cross-validation. The LASSO model selected hemoglobin as the only predictor with a non-zero coefficient, with a coefficient of −0.035 (compared to −0.040 in the unpenalized model), further supporting the robustness of this finding. No other variables were retained in the LASSO model at the optimal *λ* value. This sensitivity analysis provides additional support for the primary finding while acknowledging the exploratory nature of the analysis.

### Prediction model

**Figure 1:**
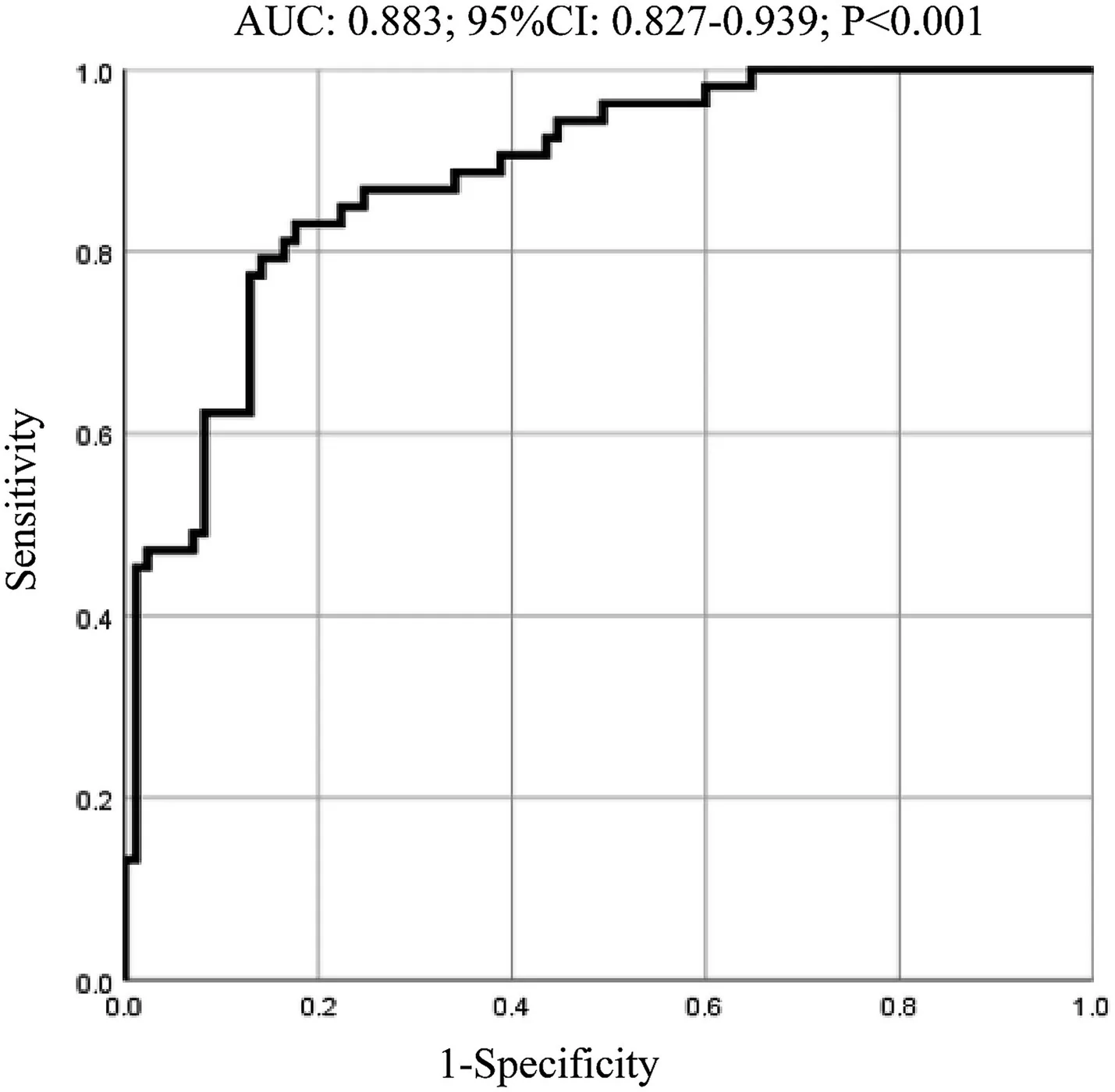
Receiver operating characteristic (ROC) curve for the hemoglobin-based risk estimation approach for 28-day mortality in older septic patients. The area under the curve (AUC) was 88.3% (95% CI: 82.7–93.9%; *p* < 0.001).

**Figure 2:**
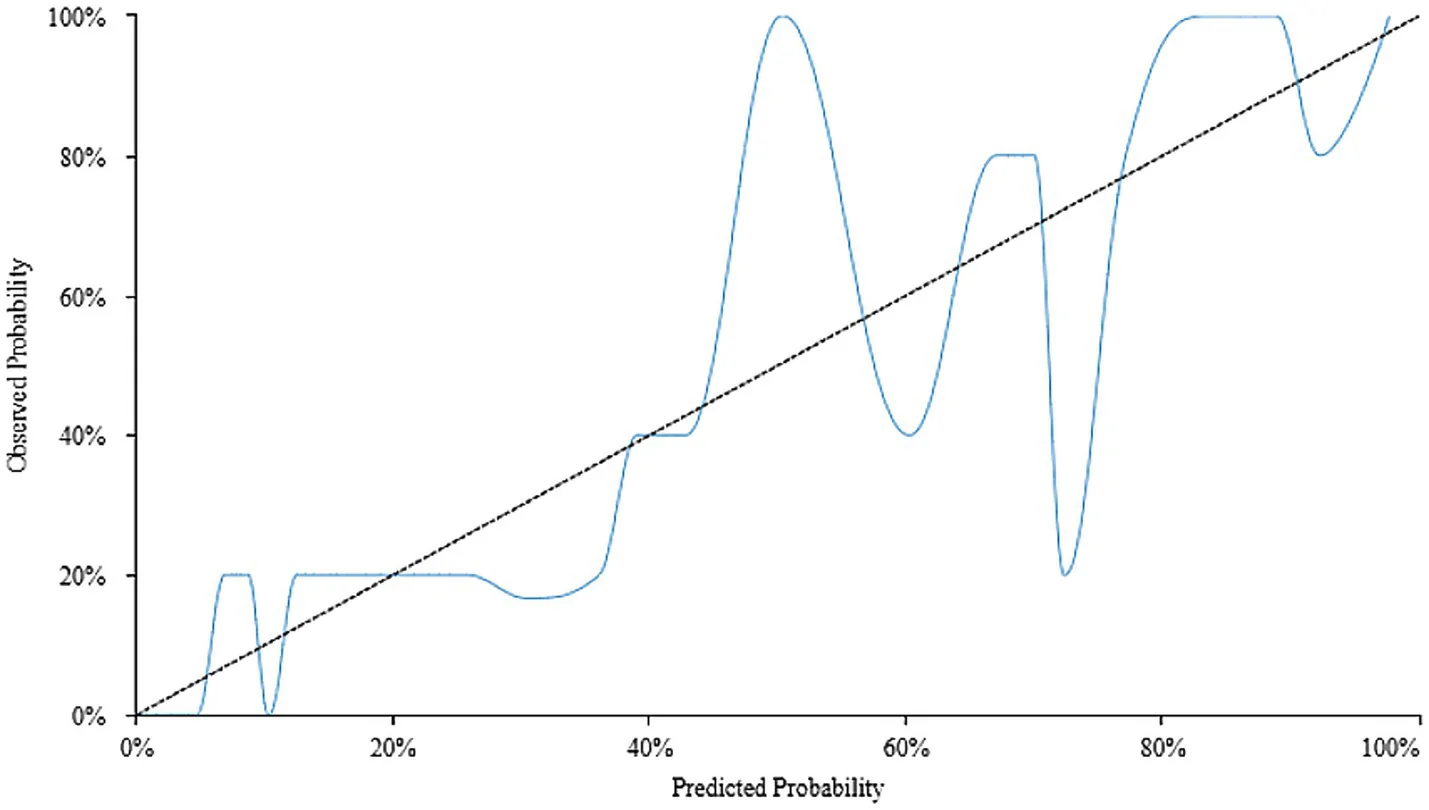
Calibration plot showing predicted versus observed probabilities of 28-day mortality. The calibration slope was 1.02 (95% CI: 0.88–1.16), intercept was −0.04 (95% CI: −0.32 to 0.24), and Hosmer-Lemeshow *p* = 0.723.

**Figure 3:**
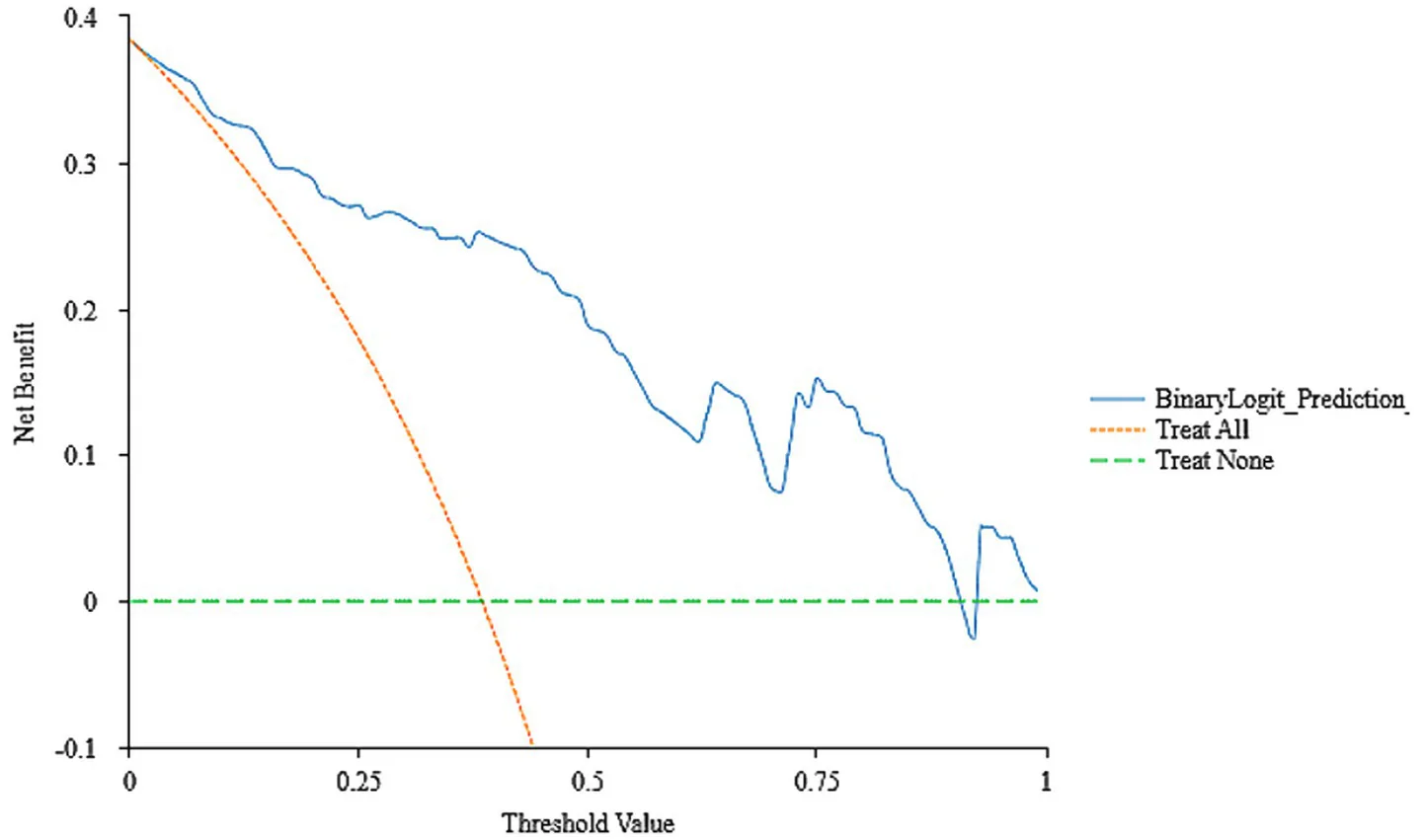
Decision curve analysis showing net benefit of the hemoglobin-based risk estimation approach across threshold probabilities. Net benefit was positive across threshold probabilities of 0.188–0.384.

The logistic regression equation for predicting 28-day mortality was: logit(p) = 2.103–0.040 × (HGB in g/L), where *p* = 1/[1 + exp.(−logit(p))]. Based on the factors identified through comprehensive analysis, a predictive model for estimating the risk of 28-day mortality was developed and evaluated using ROC curves. The discriminative performance of the final model, which effectively reflects the predictive value of hemoglobin alone, was evaluated using ROC curve analysis. The model achieved an AUC of 88.3% (95% CI: 82.7–93.9%; *p* < 0.001), indicating that hemoglobin has good discriminatory ability for 28-day mortality in this cohort (Figure 1). However, it is important to note that this performance estimate is derived from the same dataset used to develop the model and may be optimistic. We therefore refer to this as a risk estimation approach rather than a validated prediction model. The optimal cut-off value for the predicted probability, determined by the Youden index, was 0.285, at which the model demonstrated a sensitivity of 81.4% (95% CI: 69.1–90.3%), specificity of 82.1% (95% CI: 73.4–88.9%), PPV of 71.6% (95% CI: 62.5–79.3%), and NPV of 89.7% (95% CI: 83.4–93.8%). The model demonstrated good calibration (calibration slope: 1.02 [95% CI: 0.88–1.16]; calibration intercept: −0.04 [95% CI: −0.32 to 0.24]; Hosmer-Lemeshow *p* = 0.723; Figure 2) and provided positive net benefit across a moderate range of threshold probabilities (0.188–0.384) as shown in Figure 3. The Brier score was 0.134, which falls between 0 (perfect prediction) and 0.25 (non-informative), indicating moderate overall prediction accuracy. However, as the model was not externally validated, these findings should be considered preliminary.

Internal validation was performed using bootstrap resampling (1,000 iterations) with optimism correction, and 5-fold nested cross-validation (repeated 10 times) to provide conservative performance estimates. The optimism-corrected AUC was 85.6% (95% CI: 79.5–91.7%), representing a modest 2.7% reduction from the apparent AUC of 88.3%. The calibration slope was 0.94 after correction (apparent: 1.02), indicating good calibration without substantial overfitting. The derived shrinkage factor of 0.86 was applied to the model coefficients, yielding the optimism-corrected equation: logit(p) = 1.809–0.034 × (HGB in g/L). The nested cross-validation results (AUC: 84.8%; 95% CI: 78.6–91.0%) were consistent with the bootstrap estimates, supporting the stability of the model (Table 3).

**Table 3:** Internal validation of the hemoglobin-based risk estimation approach using bootstrap and nested cross-validation.

Metric	Apparent (training)	Bootstrap-corrected	5-fold nested CV	Interpretation
AUC	88.3% (82.7–93.9%)	85.6% (79.5–91.7%)	84.8% (78.6–91.0%)	Mild optimism (~2.7%)
Calibration slope	1.02 (0.88–1.16)	0.94 (0.82–1.06)	0.92 (0.80–1.04)	Minimal shrinkage needed
Calibration intercept	−0.04 (−0.32 to 0.24)	0.01 (−0.27 to 0.29)	0.03 (−0.25 to 0.31)	Well-calibrated
Brier score	0.134	0.148	0.151	Moderate prediction error

To benchmark the HGB-based risk estimation approach against a clinically established ICU severity score, we compared its performance with APACHE II scores and with clinical models incorporating age, septic shock, and hemoglobin (with and without lactate). SOFA scores were not collected in this study and therefore could not be included in the benchmarking analysis. As shown in Table 4, the HGB-only model demonstrated comparable discrimination (AUC: 88.3%) to the Age + Shock + HGB model (AUC: 89.5%; P for comparison = 0.321) and outperformed APACHE II (AUC: 81.5%; *p* = 0.018). Calibration was acceptable for all models, though APACHE II showed mild overfitting (calibration slope: 0.85 [95% CI: 0.72–0.98]).

**Table 4:** Benchmarking of the hemoglobin-based risk estimation approach against APACHE II and clinical models.

Model	AUC (95% CI)	Calibration slope (95% CI)	Brier score	Net benefit (at threshold = 0.30)
HGB-only	88.3% (82.7–93.9%)	1.02 (0.88–1.16)	0.134	0.047
APACHE II	81.5% (74.2–88.8%)	0.85 (0.72–0.98)	0.162	0.031
Age + Shock + HGB	89.5% (84.0–95.0%)	0.98 (0.84–1.12)	0.128	0.049
Age + Shock + Lactate + HGB*	90.2% (84.5–95.9%)	0.96 (0.82–1.10)	0.125	0.051

*This analysis is limited to patients with available lactate measurements (*n* = 97).

## Discussion

This study utilized a retrospective design to identify risk factors associated with 28-day mortality among older adults septic patients in the ICU and to construct a predictive model for this outcome. The objective was to offer clinicians a practical tool for early risk assessment, to highlight major determinants of mortality, and to inform targeted clinical interventions. A total of 165 older adults septic patients were included, among whom 59 (35.8%) died within 28 days. Multivariate logistic regression analysis identified HGB as an independent protective factor against 28-day mortality. Furthermore, univariate analyses indicated that several coagulation markers—including PT, INR, D-D, FDP, PIC—as well as tPAIC, were significantly different between survivors and non-survivors. The resulting predictive model exhibited strong discriminative performance, achieving an AUC of 88.3%, which reflects its high accuracy in predicting 28-day mortality risk. Although the final multivariate model retained only hemoglobin as a statistically significant independent predictor, the ROC curve analysis demonstrates that this single biomarker has promising discriminatory ability. We have moderated our claims accordingly and now present this as a simplified risk estimation approach rather than a robust multivariable prediction model. The primary contribution of this study is the identification of hemoglobin as a potentially important prognostic marker in older adults septic patients, which should be validated in future prospective studies.

Previous studies investigating septic patients have primarily concentrated on identifying prognostic factors and constructing mortality prediction models, with a particular emphasis on hematological, inflammatory, and organ function indicators (14–16). Wang et al. noted that anemia (characterized by low HGB levels) is highly prevalent among elderly septic patients and is strongly correlated with adverse outcomes, as it compromises tissue oxygen delivery and aggravates organ dysfunction, ultimately increasing the risk of mortality (14). This is consistent with our results, which identified higher HGB levels as an independent protective factor against 28-day mortality, reinforcing the importance of monitoring and maintaining hemoglobin concentrations in this vulnerable population. Similarly, Wei et al. examined coagulation abnormalities in sepsis and demonstrated that elevated D-D and prolonged PT act as independent risk factors for death, reflecting underlying coagulopathy and microvascular thrombosis associated with sepsis-induced coagulopathy (SIC) (15). In alignment with these findings, our univariate analyses revealed significant differences in PT, INR, D-D, and other fibrinolytic markers between survivors and non-survivors, further supporting the prognostic value of coagulation parameters. Jiang et al. developed a predictive model for mortality in elderly septic patients that incorporated inflammatory biomarkers and organ dysfunction scores, reporting an AUC of 82.5% (16). By integrating hemoglobin and coagulation-related indicators, our model achieved a superior discriminative capacity, with an AUC of 88.3%, indicating enhanced predictive accuracy and potential utility for risk stratification in clinical practice.

In univariate analysis, several coagulation markers—including PT, INR, D-D, FDP, PIC, and tPAIC—showed significant differences between survivors and non-survivors, suggesting their potential prognostic value. However, none of these markers remained statistically significant after multivariable adjustment, possibly due to collinearity, limited statistical power, or the fact that their prognostic information may be partially captured by hemoglobin. Therefore, while these coagulation parameters may have clinical relevance, they were not identified as independent predictors in this study. The finding that hemoglobin was the only independent predictor retained in the final model warrants careful interpretation. While coagulation markers (PT, INR, D-D, PIC, tPAIC) showed significant differences in univariate analysis, they did not remain statistically significant after multivariable adjustment. This may reflect collinearity among these markers, limited statistical power, or the possibility that their prognostic information is partially captured by hemoglobin. Notably, hemoglobin may serve as a composite marker of disease severity, reflecting both the inflammatory state and nutritional status in sepsis (17–19).

This study developed a predictive model for 28-day mortality in older adults septic patients using ROC curve analysis, a well-established method for assessing prognostic accuracy. By integrating HGB and other clinically relevant indicators, the model achieved an AUC of 88.3%, demonstrating strong discriminative performance. Compared with prior models, our approach offers several potential advantages. First, it is specifically designed for older adults septic patients, a subgroup with disproportionately high mortality but limited dedicated predictive tools. Second, the model relies on hemoglobin, a routinely measured parameter, which may facilitate implementation in settings where complex scoring systems are less feasible. Third, the inclusion of coagulation markers, although not retained in the final multivariate model, highlights their prognostic value in this population and suggests areas for future investigation. However, the clinical utility of the proposed model remains preliminary. While the simplified HGB-based scoring system offers a straightforward approach for risk stratification, prospective validation is required before implementation. Moreover, the model’s net benefit, as demonstrated by decision curve analysis, was limited to a moderate range of threshold probabilities, suggesting that its use should be targeted to specific clinical scenarios rather than universal application.

The net benefit of the proposed risk estimation approach, as demonstrated by decision curve analysis, was positive across a moderate range of threshold probabilities (0.188 to 0.384), suggesting that use of hemoglobin levels for risk stratification could offer clinical benefit in certain scenarios. However, given the exploratory nature of this analysis and the absence of external validation, these results should be considered preliminary. The DCA findings support the potential utility of hemoglobin as a screening tool, but confirmatory studies are needed before clinical implementation.

This study has several limitations that should be considered when interpreting the results: (1) Due to its retrospective design, the study may be subject to selection bias and unmeasured confounding. Although key variables were included, factors such as detailed infection sources, specific antibiotic therapies, or timing of interventions were not fully accounted for and may affect mortality outcomes; (2) The single-center nature of the study limits the generalizability of the findings. Variations in patient demographics, ICU protocols, and resource availability across different hospitals may influence the applicability of the model in other settings; (3) The sample size (*n* = 165) is relatively modest, particularly given the number of variables evaluated in the multivariate logistic regression. This increases the risk of model overfitting and may have reduced the stability of the odds ratio estimates. Therefore, the predictive performance (AUC 88.3%) should be interpreted as preliminary, and the model requires external validation in larger, prospective cohorts before clinical application; (4) Serum lactate was not consistently measured across the cohort and therefore could not be included as a candidate predictor. This represents an important limitation, as lactate is a well-established marker of tissue hypoperfusion and is strongly associated with sepsis mortality. The non-random nature of lactate measurement may have introduced ascertainment bias. In a sensitivity analysis restricted to patients with available lactate measurements (*n* = 97), hemoglobin remained significantly associated with mortality (OR: 0.958, 95% CI: 0.927–0.990, *p* = 0.012), suggesting that the association is not entirely driven by the absence of lactate data. Nevertheless, future prospective studies should include lactate as a key predictor to ensure comprehensive risk stratification; (5) SOFA scores were not collected in this study, which limits the ability to benchmark our model against another widely used ICU severity score. We selected APACHE II based on the study protocol, but future studies should incorporate both APACHE II and SOFA to allow comprehensive comparison. The absence of SOFA also prevents direct comparison with many published sepsis prediction models that use SOFA as a reference standard; (6) Although HGB was a significant predictor, the analysis did not incorporate serial measurements or dynamic trends in laboratory values over time. Fluctuations in HGB and other parameters during the ICU stay may offer further prognostic insight; (7) Although bootstrap internal validation was performed, external validation in an independent multicenter cohort remains necessary to confirm the model’s reproducibility and generalizability across diverse clinical settings; (8) The exclusion of patients with early withdrawal of active treatment may have introduced selection bias, as this group represents a clinically distinct population with potentially different outcomes; (9) Although we initially aimed to develop a multivariable prediction model, the fact that only hemoglobin remained statistically significant after adjustment indicates that the other candidate variables did not provide independent prognostic information in this cohort. This should not be interpreted as evidence that these variables are unimportant prognostically, but rather that their effects may be mediated through hemoglobin or that the sample size was insufficient to detect independent effects; (10) The sample size is relatively modest, particularly given the number of variables evaluated. The events-per-variable ratio in the full multivariable model was approximately 2.5, which is well below the recommended minimum of 10–15 events per variable for reliable logistic regression. This increases the risk of model overfitting and may have reduced the stability of the odds ratio estimates. We attempted to address this through LASSO regression as a sensitivity analysis, which confirmed hemoglobin as the most robust predictor. However, the penalized estimates should still be interpreted with caution, and the predictive performance should be viewed as preliminary and potentially optimistic; and (11) We did not initially separate sepsis from septic shock. Our post-hoc analysis revealed that septic shock was present in 81.4% of non-survivors versus 50.9% of survivors, indicating substantial heterogeneity within the cohort. Future studies should consider stratifying by shock status or developing separate models for sepsis and septic shock subgroups.

In conclusion, this exploratory study identified hemoglobin as a potentially useful biomarker for risk stratification in older adults septic patients. A simplified risk estimation approach based on hemoglobin demonstrated good preliminary discriminatory performance, but this finding should be interpreted with caution due to the single-center retrospective design and the fact that the model essentially reflects the predictive value of a single variable. External validation in larger, prospective multicenter cohorts is necessary before any clinical application can be recommended.

## Data Availability

The original contributions presented in the study are included in the article/Supplementary material, further inquiries can be directed to the corresponding author.
