# Supplementary material for: Risk factors and prediction model for mortality risk in older adults septic patients
Source: Front Med (Lausanne). 2026 Sep 1;13:1867494. doi: 10.3389/fmed.2026.1867494 (PMC13574795; doi:10.3389/fmed.2026.1867494)
Supplement: Supplementary file 1 [file Table_1.DOCX]

Supplementary Table 1. Missingness Rates per Variable and Patterns of Missing Data

| Variable | Missing n (%) | Missingness Mechanism | Reason for Missing | Measurement Availability |
| --- | --- | --- | --- | --- |
| Age | 0 (0%) | Complete | - | 100% |
| Sex | 0 (0%) | Complete | - | 100% |
| APACHE II | 0 (0%) | Complete | - | 100% |
| Hypertension | 0 (0%) | Complete | - | 100% |
| Diabetes | 0 (0%) | Complete | - | 100% |
| CVD | 0 (0%) | Complete | - | 100% |
| Stroke | 0 (0%) | Complete | - | 100% |
| Septic shock | 0 (0%) | Complete | - | 100% |
| Mechanical ventilation | 0 (0%) | Complete | - | 100% |
| Vasopressor use | 0 (0%) | Complete | - | 100% |
| Renal replacement therapy | 0 (0%) | Complete | - | 100% |
| Heparin use | 0 (0%) | Complete | - | 100% |
| HGB | 0 (0%) | Complete | Routine hematology panel | 100% |
| WBC | 3 (1.8%) | MCAR | Laboratory not ordered | 98.2% |
| Neutrophil % | 3 (1.8%) | MCAR | Laboratory not ordered | 98.2% |
| PLT | 3 (1.8%) | MCAR | Laboratory not ordered | 98.2% |
| PT | 2 (1.2%) | MCAR | Coagulation panel not ordered | 98.8% |
| INR | 2 (1.2%) | MCAR | Coagulation panel not ordered | 98.8% |
| APTT | 2 (1.2%) | MCAR | Coagulation panel not ordered | 98.8% |
| Fib | 4 (2.4%) | MCAR | Coagulation panel not ordered | 97.6% |
| CRP | 5 (3.0%) | MCAR | Laboratory not ordered | 97.0% |
| PCT | 8 (4.8%) | MCAR | Laboratory not ordered | 95.2% |
| FDP | 18 (10.9%) | MAR | Specialized assay | 89.1% |
| D-D | 15 (9.1%) | MAR | Specialized assay | 90.9% |
| TAT | 42 (25.5%) | MAR | Specialized assay | 74.5% |
| TM | 38 (23.0%) | MAR | Specialized assay | 77.0% |
| PIC | 45 (27.3%) | MAR | Specialized assay | 72.7% |
| tPAIC | 41 (24.8%) | MAR | Specialized assay | 75.2% |

MCAR: Missing Completely at Random; MAR: Missing at Random
