# Supplementary material for: Risk factors and prediction model for mortality risk in older adults septic patients
Source: Front Med (Lausanne). 2026 Sep 1;13:1867494. doi: 10.3389/fmed.2026.1867494 (PMC13574795; doi:10.3389/fmed.2026.1867494)
Supplement: Supplementary file 2 [file Table_2.DOCX]

Supplementary Table 2. Sensitivity Analyses for Informative Missingness in Specialized Coagulation Assays

| Imputation/Sensitivity Strategy | Analysis Population | HGB OR (95% CI) per 10 g/L | HGB P value | Model AUC (95% CI) | Conclusion |
| --- | --- | --- | --- | --- | --- |
| Primary Analysis |  |  |  |  |  |
| Complete case analysis (exclude patients with missing TAT/TM/PIC/tPAIC) | n = 120 | 0.651 (0.509-0.832) | 0.001 | 87.9% (81.0-94.8%) | Reference |
| Multiple Imputation Approaches |  |  |  |  |  |
| MICE imputation (20 imputations)* | n = 165 | 0.661 (0.534-0.818) | <0.001 | 88.1% (82.5-93.7%) | Consistent |
| Predictive mean matching imputation | n = 165 | 0.658 (0.529-0.819) | <0.001 | 88.0% (82.4-93.6%) | Consistent |
| MNAR Scenario Modeling |  |  |  |  |  |
| Missing values imputed +1 SD (worse outcomes assumed) | n = 165 | 0.655 (0.520-0.825) | <0.001 | 87.6% (81.8-93.4%) | Consistent |
| Missing values imputed -1 SD (better outcomes assumed) | n = 165 | 0.663 (0.535-0.822) | <0.001 | 87.8% (82.0-93.6%) | Consistent |
| Pattern Mixture Models |  |  |  |  |  |
| Stratified by TAT missingness (missing vs. observed) | n = 165 | 0.662 (0.533-0.823) | <0.001 | 87.7% (81.9-93.5%) | No interaction (P=0.218) |
| Stratified by PIC missingness (missing vs. observed) | n = 165 | 0.660 (0.531-0.820) | <0.001 | 87.8% (82.0-93.6%) | No interaction (P=0.305) |
| Restricted Subgroup Analyses |  |  |  |  |  |
| Patients with all coagulation markers available | n = 97 | 0.648 (0.498-0.843) | 0.001 | 88.5% (81.0-96.0%) | Consistent |
