# Supplementary material for: Risk factors and prediction model for mortality risk in older adults septic patients
Source: Front Med (Lausanne). 2026 Sep 1;13:1867494. doi: 10.3389/fmed.2026.1867494 (PMC13574795; doi:10.3389/fmed.2026.1867494)
Supplement: Supplementary file 3 [file Table_3.DOCX]

Supplementary Table 3. Subgroup analyses according to patients’ age.

| Characteristic | Age 55-64 (n=54) | Age ≥65 (n=111) | P for interaction |
| --- | --- | --- | --- |
| N (%) | 54 (32.7%) | 111 (67.3%) | - |
| 28-day mortality | 17 (31.5%) | 42 (37.8%) | 0.424 |
| HGB (mean±SD) | 94.2 ± 24.1 | 96.8 ± 24.5 | 0.523 |
| HGB OR (per 10 g/L) | 0.702 (0.518-0.951) | 0.651 (0.509-0.832) | 0.712 |
| AUC (HGB model) | 86.5% (75.8-97.2%) | 89.1% (82.4-95.8%) | 0.683 |
